# The Current Role of Stem Cells in Orthopaedic Surgery

**DOI:** 10.5704/MOJ.1511.016

**Published:** 2015-11

**Authors:** HH Maniar, AA Tawari, M Suk, DS Horwitz

**Affiliations:** Department of Orthopaedic Surgery, Geisinger Medical Center, Danville, USA

**Keywords:** Stem cell, orthopaedic surgery

## Abstract

Basic science and experimental research on stem cells has increased exponentially in the last decade. Our present knowledge about stem cell biology is better than ever before. This new paradigm shift in research has been reflected in the field of orthopaedic surgery. Various experimental models have suggested a potential application of stem cells for different orthopaedic conditions, and early clinical results of stem cell use have been encouraging. These cells can be easily isolated, processed and made available for clinical use. From healing of bone defects caused by trauma, tumor or infection to cartilage defects, nerve, tendon and ligament healing, stem cell use has the potential to revolutionize orthopaedic practice. The purpose of this article is to orient a general orthopaedic surgeon towards the current use and clinical applications of stem cell based therapy in orthopaedics and to provide a complete overview of the clinical advances in this field.

## Introduction

With an increased emphasis on evidence based medicine, there has been an increasing focus on the pathophysiology of orthopaedic injuries and disease processes, and their impact on overall outcome. Traditional treatment strategies are evolving to encompass tailored approaches that account for age, occupation and patient expectation. Newer strategies for management are routinely implemented and encouraged in an effort to improve patient outcomes. It is to achieve these objectives that regenerative medicine which utilizes the use of stem cells and tissue engineering has newly emerged.

### Definitions:

Stem Cells: Undeveloped biological cells capable of proliferation, self-renewal, conversion to differentiated cells and regenerating tissues^1^.

Totipotent cells: These are cells that can develop into all cell types in the human body and can also form extra embryonic and placental cells. The cells of the early stages of the embryo are the only totipotent cells and are not in clinical use due to ethical concerns^2^.

Pluripotent cells: Cells that can develop into cells of all the three germ layers (endoderm, ectoderm or mesoderm). Cells of late stages of embryo after the blastocyst stage are pluripotent cells^2^.

Multipotent cells: These are cells that can develop into more than one but not all germ layers. Adult stem cells and cord blood cells are multipotent cells^2^.

### Types of Stem Cells:

Embryonic Stem Cells (Pre-natal): These cells are obtained from the blastocyst stage of the embryo. Pluripotent in the truest sense, they have a capacity to form into any tissue of the body and multiply in an unlimited manner. This is predominantly due to the phenomenon of asymmetric division – production of one stem and one non-stem daughter cell^3^. These properties, however, also make them prone to tumorigenesis^4^. This and the necessity of harvest from embryos causes safety and ethical dilemma.

Adult Stem cells (Post-natal): These cells are obtained later in life after the embryonic stage. They are multipotent, undifferentiated cells located among specialized tissues with a primary function of their maintenance and repair. Mesenchymal stem cells (MSC), which originate from the mesoderm, are a type of adult stem cells that have a good potential to develop into adipocytes, chondrocytes, myoblasts and osteoblasts.

### Sources of Stem cells:

Stem cells can be obtained from bone marrow, periosteum, adipose tissue, placenta, umbilical cord, blood, human amniotic fluid, dental pulp, synovial tissue, skin and skeletal muscle. Among these, bone marrow, adipose tissue and muscle derived MSCs are most commonly used as they are easily obtained and abundantly available^1^.

### Isolation of Stem cells:

Stem cells may be “unselected cells” obtained from autologous bone marrow after centrifugation or “selected” and enhanced in culture utilizing their affinity to tissue plastics. Cost involved, time to culture, risk of infection and loss of function *in vitro* are factors preventing regular clinical use of cultured MSCs. It should be noted that absolute number and the purity of cells obtained from cultures is higher, an important factor for clinical effect^5^. The posterior iliac crest has been shown to have a higher yield for MSCs as compared to anterior in case of bone marrow aspiration^6^.

### Route of administration:

Stem cells may be directly applied into a lesion either surgically or via local injection with a suitable scaffold/carrier. MSCs may be taken through initial phases of differentiation, forming bone or cartilage precursors under laboratory conditions and then implanted into lesions. In addition, MSCs may be administered intravenously. Their ability to migrate systemically and colonize the bone marrow after a peripheral injection has been utilized for treatment of Osteogenesis Imperfecta^7^.

### Mechanism of action:

In addition to differentiation into bone, muscle, cartilage, ligament or tendon cells, MSCs also have a paracrine effect whereby they secrete growth factors and cytokines such as bone morphogenic proteins (BMPs), transforming growth factor-β (TGF-β), and vascular endothelial growth factor (VEGF). These play an important role in angiogenesis, repair, cell survival and proliferation. MSCs also have the ability to migrate to the site tissue injury to modulate an inflammatory response^8^. Genetically modified MSCs for long term release of growth factors are being currently developed.

### Role of Mesenchymal Stem Cells in Orthopaedic Surgery:

MSCs have an ability to develop into any mesodermal tissue. Thus, they can be prompted to form precursor cells to develop into tissues including bone, cartilage, muscle, tendon, and ligament. The use of stem cells for various orthopaedic challenges is outlined below.

### Trauma and bone defects:

Nonunion/Delayed union and bone defects following trauma, tumor or infection are challenging aspects of orthopaedic surgery that may require biologic augmentation for optimum healing. Autologous cancellous graft is the current ‘gold standard’, but limited supply and donor site morbidity limit their use. Allografts and bone graft substitutes are routinely used to augment bone healing. However, poor graft integration and osteonecrosis of the graft remain primary issues with this method. Bone marrow aspirates that contain stem cells in a proportion of 1:10,000 to 1:1,000,000 of nucleated cells have been successfully used to enhance healing of non-unions^5^. Tissue engineering, involving the use of stem cells with scaffolds such as hydroxyapatite (HA), demineralized bone matrix (DBM) and tri-calcium phosphate (TCP), have been studied and found to be useful for bridging bone defects^9^. Due to absence of an extracellular matrix to grow on, MSCs alone have not proven to be beneficial for filling defects caused by simple/aneurysmal bone cysts^10^. Healing rates, are however, enhanced when these are used in conjunction with scaffolds.

### Spine and peripheral nerve surgery:

#### Spine Fusion:

Neen *et al*, in a prospective study, showed that unselected stem cells used with HA scaffolds had similar healing rates as autologous grafting; thereby preventing donor site morbidity^11^. Similar results were obtained by Gan et al using β-TCP^12^.

#### Intervertebral Disc Degeneration:

Intervertebral disc degeneration is one of the most common causes of backache in a young productive population. Despite the high prevalence there is no treatment available which reverses the primary pathology. Animal experiments have shown increased proteoglycan content and maintenance of disc height with percutaneous stem cell injections^13^. Clinical trials are in progress to evaluate these results in humans with positive interim results^14^.

#### Spinal cord and peripheral nerve injuries:

Spinal cord and peripheral nerve injuries have a significant impact on quality of life of affected individuals. Animal studies have highlighted some positive effects of MSC use via intrathecal and local administration, however, the response seen in clinical studies is mixed^15^. In an animal study, Tamaki *et al*, demonstrated that muscle derived MSCs aided in successful regeneration of a crushed peripheral nerve^16^. Further prospective clinical studies are necessary to establish the role of MSCs in managing these patients.

### Articular cartilage:

Articular cartilage is a highly specialized tissue with a poor intrinsic capacity to repair itself. The goal of any cartilage procedure is to restore its integrity so that it can withstand the wear and tear of daily activity.

#### Focal cartilage damage:

Since Pridie introduced subchondral drilling in the late 1950s, various procedures such as microfracture and abrasionplasty have been developed to recruit MSCs from adjacent bone marrow to proliferate into chondrocytes. Unfortunately, these procedures result in the formation of an inferior quality nonhyaline cartilage. Data on use of MSCs with suitable scaffolds in cartilage healing is mostly based on animal studies, with a few human case series showing improved healing and better function after autologous MSC implantation techniques^17^.

#### Osteoarthritis (OA):

Due to their role in inhibiting the catabolic activity of matrix metalloproteinases (MMP), MSCs have been shown to have a beneficial effect in OA^18^. In a recent study, Sato *et al* showed that guinea pigs with age related OA treated with MSC laden hyaluronic injections had better cartilage regeneration with higher type II collagen and lower MMP content^19^. Except for a few case series which show some clinical improvement there is a paucity of trials with human subjects which study the effect of MSCs on OA^20^.

#### High tibial Osteotomy (HTO) and Arthoplasty:

In a randomized control trial, Dallari *et al* showed that lyophilized bone chips treated as grafts with platelet gels and MSCs had higher rate of osteointegration in HTOs^21^. With appropriate use of nanotechnology to make optimum implant surfaces, MSCs have a great potential to revolutionize joint replacement surgery by facilitating osteointegration. Three dimensional scaffolds with MSCs may be used in the future to form autologous osteochondral grafts suitable for a ‘biologic’ arthroplasty^22^.

### Avascular Necrosis:

Avascular Necrosis (AVN) of the head of femur is one of the most debilitating disorders in young patients. It is characterized by a decreased blood supply to the bone and associated increase in intraosseous pressure. The integrity of the subchondral plate is one of the most important deciding factor between head preserving (core decompression, bone grafting, femoral osteotomies) or head sacrificing (hip resurfacing/arthroplasty) procedures. Stem cells have angiogenic and osteogenic properties. Early stages of AVN are amenable to treatment with stem cell concentrate injection combined with routine retrograde procedures such as core decompression. Bone marrow aspirates administered after core decompression have been shown to be beneficial in AVN. Stem cells were isolated and used in a study by Rastogi *et al* where 60 hips in early stages of AVN were randomized to be treated either with core decompression and bone marrow injection or with core decompression and injection of isolated stem cells^23^. Two year follow up showed a better functional outcome and better radiographic healing in the stem cell group.

### Wound Healing:

Although not typical in orthopaedic practice, poorly healing wounds are commonly encountered in treating patients with risk factors such as diabetes or open fractures. MSC treatment of acute and chronic wounds results in accelerated wound closure with increased epithelialization, granulation tissue formation and angiogenesis^24^.

### Bone-Tendon interface and Tendon Healing:

Numerous commonly employed surgical procedures such as anterior cruciate ligament reconstruction, rotator cuff repair or retrocalcaneal bursa excisions depend on optimum healing of the bone-tendon interface. Fibrovascular scar formed during healing possesses inferior biochemical and mechanical properties. MSCs have been shown to promote early healing of the bone tendon interface by increasing the proportion of Sharpey’s fibers. MSCs used with bone morphogenic protein 2 (BMP-2) are associated with improved biomechanical properties of the bone tendon interface including stiffness and maximal load. A recent study by Adams *et al* showed that rats with Achilles tendon tear treated with stem cell-bearing sutures have higher failure strength and better histological properties^25^. Unselected MSCs were used for ultrasound-guided injections in a case series by Pascual-Garrido *et al* for chronic patellar tendinopathy with good clinical results^26^.

### Paediatric Orthopaedics:

#### Osteogenesis Imperfecta (OI):

This is a heterogenous group of diseases with abnormality of type I collagen primarily leading to increased susceptibility to fractures, slow growth and loss of bone mass. Systemic infusion of allogenic MSCs by Horwitz *et al* in six children with OI showed improvement in bone mass and bone growth acceleration^7^.

#### Physeal injuries:

Bone bridge formation is an adverse complication following traumatic, infectious or other insult on the physis, leading to angular and/or longitudinal deformities. In a pig study, Planka *et al* showed that MSCs with scaffolds used in physeal defects differentiated into chondrocytes forming hyaline cartilage and prevented bony bridge formation^27^. Currently, there are no clinical studies to support this.

### Osteoporosis:

In spite of tremendous advances in drug therapy, osteoporosis plays a significant role in overall health of geriatric patients. Increasing age is associated with decrease in number and function of osteoblasts and osteoprogenitor cells. Systemic infusion of MSCs has failed to promote bone formation due to their inability to migrate to the surface of the bone, a critical step for bone formation. In an animal study, Guan *et al* used MSCs modified to express certain surface proteins which enabled them to migrate to the periosteum leading to increased trabecular bone formation and bone mass^28^. Concepts such as these are positive steps towards utilizing MSCs for a generalized disease like osteoporosis.

### Muscular dystrophies:

These are group of conditions wherein muscle fibers are replaced by fibrotic and adipose tissues, due to genetic mutations in several muscle proteins, which are essential for normal muscle function. There is no cure for these patients and treatment is focused on comfort care, respiratory assistance and delaying loss of function. Local and systemic transplantation of well differentiated myoblasts is associated with poor cell survival, limited migration from injection site and immune rejection. In a phase I clinical trial, Torrente et al showed that muscle derived stem cells with specific surface markers were safely transplanted in eight boys with no side effects^29^. Genetically modified MSCs are being developed for potential use of these cells in Muscular dystrophies.

### Summary:

Regenerative medicine with the use of stem cells is expected to revolutionize patient treatment. Their utilization for bone tissue engineering with appropriate scaffolds provides us with exciting opportunities for research and development. Efforts must be made to ensure safe, economical, efficient and effective introduction of stem cells for regular clinical use. Well-developed, randomized, prospective, clinical studies that build on the existing animal data will provide us with much needed information for the correct application of this field of science to well documented needs of orthopaedic surgeons. (Table I summarizes all recent clinical studies utilizing stem cell use in orthopaedic surgery organized by levels of evidence)

**Table I:** Recent (within 5 years) clinical studies (minimum 10 patients) utilizing stem cell use in orthopaedic surgery, sorted by levels of evidence

No	Type of MSC	Study Type	Pathology	No of Patients	Results	Reference	Follow up
1	Cultured stem cells obtained from skin fibroblasts and modulated to grow collagen producing cells	Level I – Randomized Control Trial	Patellar tendinopathy	46 patients, 60 patellar tendons	Improvement in pain and function.	Clarke AW et al., [30] 2011	6 months
2	Cultured cells differentiated into osteoblasts injected with fibrin	Level II- Randomized Control Trial with no blinding	Fracture healing rate and safety	64	Acceleration of fracture healing and safe	Kim SJ et al., [31] 2009	2 months
3	Uncultured mononuclear cell instillation obtained from bone marrow instilled following core decompression	Level II – Prospective comparative study	Avascular Necrosis of head of femur	40 patients, 51 hips	Better clinical scores and radiological outcomes	Sen RK et al., [32] 2012	2 years
4	Uncultured cell instillation obtained from bone marrow and implanted with core decompression	Level II – Prospective comparative study	Avascular Necrosis of head of femur	19 patients, 24 hips	Improvement in pain and progression to collapse	Gangji V et al., [33] 2011	5 years
5	Cultured cells transplanted to are a surrounding injury	Level III – Case Control study	Complete and Chronic Cervical Spinal cord injury	40 patients. 20 cases and 20 controls	Improvement in neurological function at 6 months in 10/20 patients in treatment group	Dai G et al., [34] 2013	6 months
6	Cultured bone marrow derived stem cells	Level III – Prospective comparative	Articular cartilage repair	72	No significant improvent as comparted to autologous chondrocyte implantation except for one stage procedure.	Nejadnik H et al., [17] 2010	24 months
7	Uncultured bone marrow derived stem cells with hyaluronic acid membrane scaffold with platelet rich fibrin	Level III – prospective comparative	Osteochondral lesions of Knee	20	No significant improvent as comparted to autologous chondrocyte implantation except for one stage procedure	Buda R et al., [35] 2010	2 years
8	Uncultured mononuclear cell concentrate obtained from peripheral blood and reinfused through arteriography	Level IV – Case Series	Chronic Spinal Cord Injuries	39	26/39 (66.7%) patients showed recovery of somatosensory evoked potentials	Cristante AF et al., [36] 2009	2.5 years
9	Uncultured cells obtained from	Level IV -Case Series	Knee cartilage repair	52	Improvement in Knee scores	Skowronski J et al., [37] 2012	6 years
10	Cultured – Collagen producing stem cells obtained from skin	Level IV – Case series	Lateral Epicondylitis	12	Improvement in elbow scores and chondrocyte showing features of healing	Connell D et al., [38] 2009	6 months
11	Uncultured cells implanted arthroscopically with collagen powder or Hyaluronic acid scaffolds with platelet gel	Level IV -Therapeutic Study	Osteochondral lesions of talus	48	Improvement in Ankle Score. Histologically, tissue in various degrees of remodeling but none was entirely hyaline cartilage	Giannini S et al., [39] 2009	Minimum 2 years, Mean 29 months
12	Uncultured mononuclear cells obtained from bone marrow aspiration with collagen sponge scaffold	Level IV -Case Series	Filling of Bone defects. (Trauma and Tumor)	10	Healing of all bone defects	Jager M et al., [40] 2009	Variable (maximum -18 months
